# Influence of experiencing SARS-CoV-2 infection on anxiety levels in Chinese patients undergoing third molar surgery

**DOI:** 10.3389/fpsyg.2024.1307776

**Published:** 2024-03-21

**Authors:** Junfei Zhu, Wenjing Li, Fang Wei, Dan Zhang, Meng Wang, Huiyun Zhang, Ye Zhang

**Affiliations:** Stomatology Center of China Japan Friendship Hospital, Beijing, China

**Keywords:** COVID-19, third molar, dental fear, MDAS questionnaire, anxiety

## Abstract

**Background:**

In China, most of the citizens experienced SARS-CoV-2 infection since the end of 2022. The Coronavirus disease 2019 (COVID-19) pandemic affected people’s physical health and also had a significant impact on mental well-being. The present study aims to discover if the experience of SARS-CoV-2 infection influences patients’ anxiety toward third molar surgery in the Chinese population.

**Materials and methods:**

The present study took the form of a questionnaire survey. From January 1, 2023, to June 30, 2023, patients who went to the Stomatology Center of China-Japan Friendship Hospital (Beijing, China) for the third molar extraction were included according to the inclusion criteria. The information on COVID-19 infection and the Modified Dental Anxiety Scale (MDAS) was collected. The software SPSS 22.0 was used for the statistical analyses.

**Results:**

A total of 574 survey results were harvested in the present study. The infection rate of COVID-19 was 86.6% (*p* > 0.05). The Average MDAS scores between patients who had been infected with COVID-19 and patients who were never infected were not significantly different (11.65 ± 4.41 vs. 11.42 ± 4.41, *p* > 0.05). The subgroup analysis was conducted according to the length of time after the recovery of COVID-19 (Model 1), and the highest temperature during the infection (Model 2). In Model 1 and Model 2, the one-way ANOVA test did not find statistical significance between the groups (Model 1 *p* = 0.114; Model 2 *p* = 0.481). The MDAS scores in female patients were significantly higher than in male patients (12.29 ± 4.53 vs. 9.91 ± 3.80, *p* < 0.001). Patients who extracted double teeth got significantly higher MDAS scores than those who extracted single teeth before the surgery (12.03 ± 4.74 vs. 11.24 ± 4.18, *p* = 0.037).

**Conclusion:**

The present study did not establish a significant impact of SARS-CoV-2 infection on the anxiety levels associated with third molar surgery among Chinese patients. The potential long-term biopsychological effects of the virus warrant further investigation.

## Introduction

The global burden of Severe Acute Respiratory Syndrome Coronavirus 2 (SARS-CoV-2) impacted populations worldwide (Wang et al., 2023). Evidence from previous coronavirus outbreaks has shown that the Coronavirus disease 2019 (COVID-19) pandemic has not only brought about health concerns and economic disruptions but has also led to a significant increase in anxiety among infected patients worldwide (Zhang et al., 2022). COVID-19 anxiety refers to the heightened worry, fear, and stress experienced as a result of the pandemic. It encompasses a range of concerns, including fears about personal health, uncertainty about the future, social isolation, and the overwhelming amount of information surrounding the virus. Acknowledging and addressing COVID-19 anxiety is crucial for our overall well-being during that challenging time (Jones et al., 2021).

Dental anxiety refers to the fear or nervousness that individuals experience when faced with dental appointments or procedures. The reported preoperative anxiety rate of dental patients is up to 60 to 80% (Sancak and Akal, 2019). Third molar surgery, also known as wisdom tooth extraction, is the most common dental surgery done by oral and maxillofacial surgeons (Sifuentes-Cervantes et al., 2021). The surgery should be proceeded when the third molar is symptomatic or there is evidence of disease regarding the third molar (Steed, 2014). The association between third molar extraction and anxiety is a topic of great significance in the field of dentistry (Souza et al., 2022). The COVID-19 pandemic has heightened general health anxieties as well as mental anxieties (Jafri et al., 2022). This concern is particularly relevant to third molar surgeries. The overall increase in stress and anxiety levels due to the pandemic can have a spill-over effect on how patients perceive and experience dental treatments (Nikolić et al., 2022). People who are already dealing with heightened anxiety or stress may find it more challenging to cope with the prospect of undergoing third molar surgery.

During the initial phase of the pandemic, China implemented a dynamic zero-COVID strategy, focusing on controlling sources of infection, interrupting transmission pathways, and safeguarding vulnerable populations (Bai et al., 2022). On December 7, 2022, in response to the evolving characteristics of COVID-19 variants and changing epidemic conditions, China released the “Notice on Further Optimizing the Implementation of COVID-19 Prevention and Control Measures,” signaling a strategic shift away from the stringent zero-COVID policy (Song et al., 2023). This abrupt policy change led to significant impacts, including overwhelmed healthcare facilities and funeral homes, as well as a historically high youth unemployment rate (Li et al., 2023; Mallapaty, 2023). During the COVID-19 pandemic, dentists and oral surgeons have been taking extra precautions when it comes to handling procedures involving third molars. Therefore, a large amount of third molar extraction surgeries were postponed (Jin et al., 2023). After the emergence of the infection, people who recovered from the disease started to accommodate the post-pandemic lives, and go to the dental clinics and hospitals to receive the third molar surgeries.

Strict lockdowns measures and intense public health messaging have led to a deep fear of COVID-19 among Chinese people. Then, the country experienced a rapid surge in COVID-19 cases and deaths, and most of the citizens experienced SARS-CoV-2 infection since the end of 2022. After such an experience, the hypothesis of the present study is that patients who have contracted COVID-19 might undergo several psychological changes. These changes could potentially influence the level of anxiety experienced by citizens regarding dental treatments. In the present study, we collected the post-epidemic patients’ Modified Dental Anxiety Scale (MDAS) scores toward the third molar surgeries, and the aim is to discover if the experience of SARS-CoV-2 infection influences patients’ anxiety toward third molar surgery.

## Materials and methods

### Study design

The present cross-sectional study took the form of a questionnaire survey. The survey was performed from January 1, 2023, to June 30, 2023. The study population consisted of patients referred to the Department of Oral and Maxillofacial Surgery of the Stomatology Center of China-Japan Friendship Hospital (Beijing, China) for the third molar extraction. The inclusion criteria consisted of (1) Patients with no history of mental illness, (2) Patients who are fully recovered from the covid-19 or patients who are never infected with the virus, and (3) Patients who have no contraindication to third molar surgery.

Patients who satisfying the inclusion criteria were assigned the questionnaires before surgery. The questionnaire consisted of the following items: (1)Age; (2)Gender; (3) Have you been infected with Covid-19; (4) How long since you recovered from the infection; (5) The highest body temperature during the infection, and (6) the MDAS. Patients who never experienced COVID-19 symptoms and have never been detected with COVID-19 positive (Including antigen, antibody, and nucleic acid) are considered of no history of COVID-19 infection.

The MDAS was first designed in 1995 (Humphris et al., 1995). Five items were included in the questionnaire, and five responses were provided under each item of the MDAS, rising from “not anxious” to “extremely anxious,” with accompanying scores ranging from 1 to 5 (Shacham et al., 2022). Therefore, the total scores of the MDAS are between 5 to 25.

### Statistical analysis

The software SPSS 22.0 was used for the statistical analyses. The data of continuous variables were presented as mean ± standard deviations. When comparing two groups, we employed the Student’s t-test, a widely used method to determine if there is a statistically significant difference between the average values of the groups. For comparisons involving more than two groups, we used the One-way Analysis of Variance (ANOVA), which tests for significant differences in the mean values across multiple groups controlled by a single independent variable. Furthermore, we applied Pearson’s correlation analysis to assess the strength and direction of the linear relationship between the Modified Dental Anxiety Scale (MDAS) scores and other continuous variables, allowing us to understand how these variables may relate to dental anxiety. The level of statistical significance was fixed at *p* < 0.05.

## Results

### Characteristics

**Figure 1:**
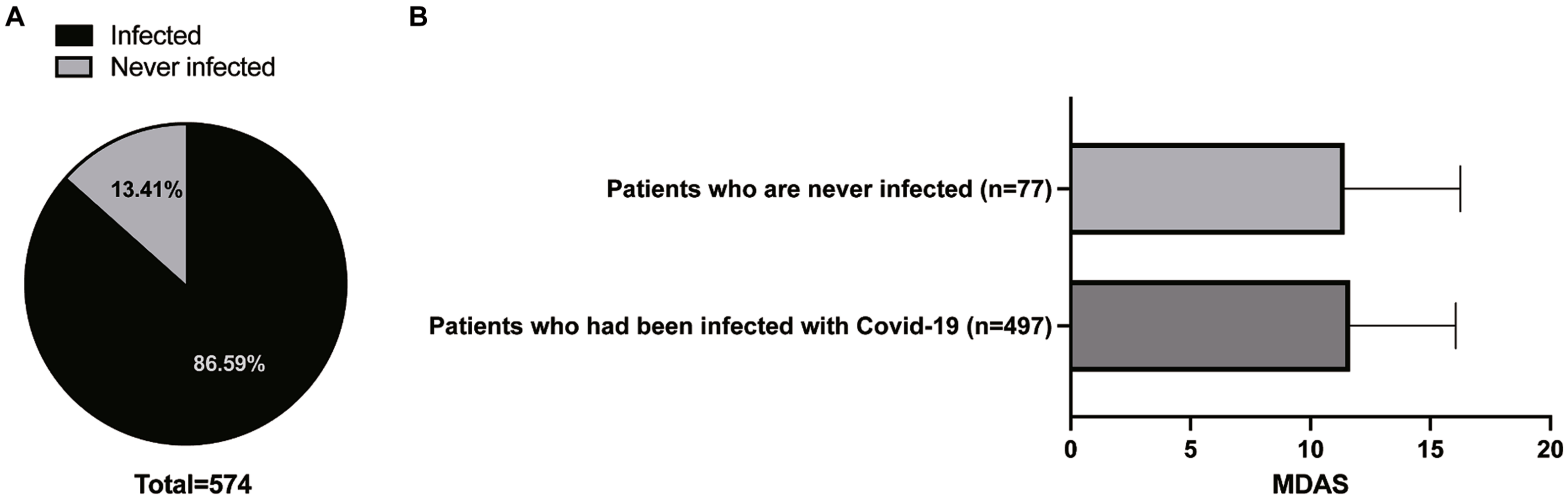
The infection rate of COVID-19 **(A)** and the Average MDAS scores of patients who had been infected with COVID-19 and who were never infected **(B)**.

A total of 624 surveys were distributed to patients, of which 50 were either refused or failed to complete the survay due to patients’ inability to recall detailed information. Consequently, 574 surveys were effectively collected for the present study, resulting in a response rate of 92%. The Average age was 28.36 ± 6.57, ranging from 12 to 67 years. 411 (71.6%) patients were female and 163 (28.4%) were male. Of the total, 497 patients experienced the infection of COVID-19 with the average highest temperature of 38.57 ± 0.76, and 77 patients do have a history of COVID-19 infection. The infection rate was 86.6%. The Average MDAS scores of patients who had been infected with COVID-19 and patients who were never infected were 11.65 ± 4.41 and 11.42 ± 4.41saperately, which showed no statistical significance (*p* > 0.05) (Figure 1).

The subgroup analysis was conducted, and the data was grouped according to the length of time after the recovery of Covid-19 (Model 1), and the highest temperature during the infection (Model 2). The characteristics of Model 1 and Model 2 are presented in Tables 1, 2.

**Table 1:** Charicteristcs of the population when grouping by the length of time after the fully recovery from COVID-19 (Model 1).

	1–2 months	2–3 months	3–4 months	4–5 months	5–6 months	Negtive
Groupe size	201	60	126	51	59	77
Gender (Female/male)	134/67	47/13	99/27	34/17	39/20	58/19
Age	28.86 ± 6.86	27.32 ± 5.30	27.97 ± 5.96	29.69 ± 7.97	29.02 ± 7.77	27.18 ± 5.36
Highest body temperature	38.53 ± 0.81	38.78 ± 0.69	38.62 ± 0.76	38.62 ± 0.70	38.35 ± 0.71	NA
The number of extracted tooth	1.46 ± 0.50	1.5 ± 0.50	1.5 ± 0.50	1.49 ± 0.54	1.58 ± 0.50	1.38 ± 0.49
Sides(Maxillary/mandible/both)	28/79/94	9/22/29	8/56/62	7/19/25	3/22/34	5/43/29
Sides(Left/right/both)	97/103/1	29/30/1	62/63/1	30/21/0	36/23/0	44/33/0
MDAS	11.17 ± 4.26	12.25 ± 4.54	12.01 ± 4.68	12.24 ± 5.30	11.30 ± 3.53	11.43 ± 4.82

**Table 2:** Charicteristcs of the population when grouping by the highest body temperature during COVID-19 (Model 2).

	Negtive	<37°C	37–38°C	38–39°C	39–40°C	≥40°C
Groupe size	77	14	57	231	180	15
Gender (Female/male)	58/19	8/6	38/19	168/63	126/54	13/2
Age	27.18 ± 5.36	31.00 ± 9.08	27.93 ± 7.07	29.27 ± 7.39	27.90 ± 5.50	25.67 ± 3.33
The number of extracted tooth	1.38 ± 0.49	1.50 ± 0.52	1.55 ± 0.50	1.47 ± 0.50	1.50 ± 0.50	1.47 ± 0.52
Sides(Maxillary/mandible/both)	5/43/29	4/3/7	5/20/32	23/102/106	22/67/91	1/7/7
Sides(Left/right/both)	44/33/0	4/10/0	35/22/0	116/113/2	92/87/1	7/8/0
MDAS	11.43 ± 4.82	9.5 ± 3.53	12.07 ± 4.89	11.63 ± 4.16	11.62 ± 4.56	12.53 ± 4.97

**Figure 2:**
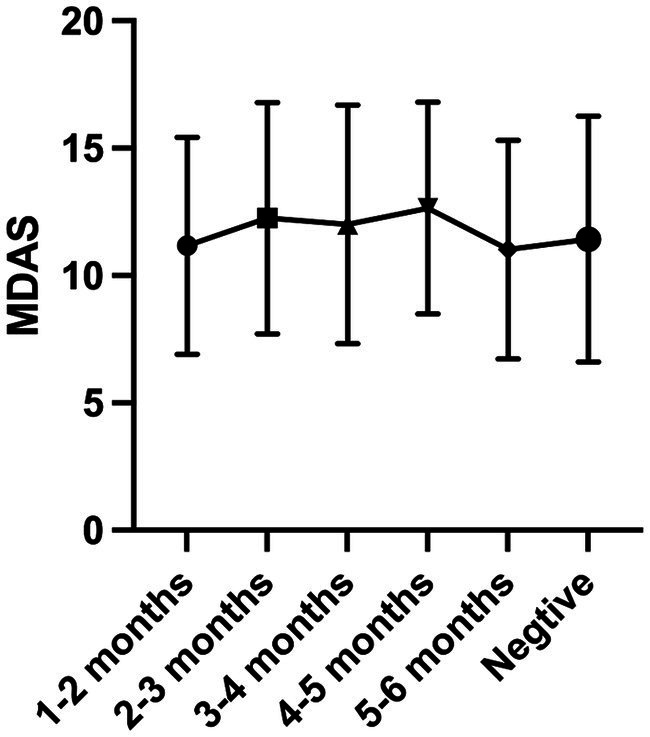
The comparison of the average MDAS scores between the groups of Model 1.

**Figure 3:**
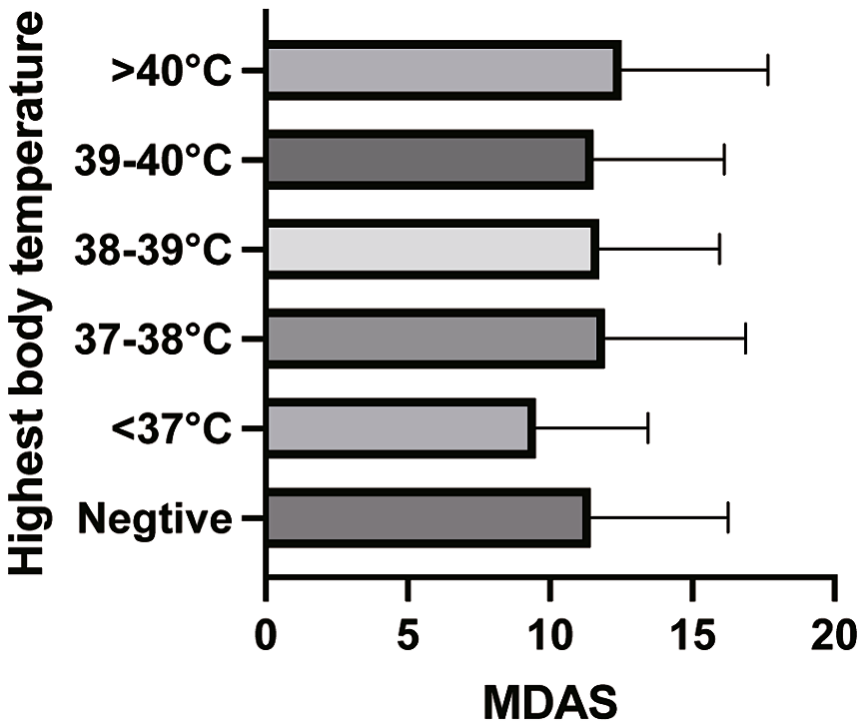
The comparison of the average MDAS scores between the groups of Model 2.

In Model 1 and Model 2, the one-way ANOVA test did not find statistical significance between the groups (Model 1 *p* = 0.114; Model 2 *p* = 0.481) (Figures 2, 3).

In addition, we found the MDAS scores in female patients were significantly higher than in male patients (12.29 ± 4.53 vs. 9.91 ± 3.80, *p* < 0.001). And, we also found patients who extracted double teeth got significantly higher MDAS scores than those who extracted single teeth before the surgery (12.03 ± 4.74 vs. 11.24 ± 4.18, *p* = 0.037).

Through the Pearson Correlation analysis, we found that MDAS scores were negatively correlated with age (*p* = 0.025). And, The highest temperature during infection and MDAS scores were not correlated (*p* = 0.289).

## Discussion

This study gathered data on patients’ Modified Dental Anxiety Scale (MDAS) scores in relation to third molar surgery following their recovery from COVID-19. Our findings indicate that a history of COVID-19 infection does not significantly affect patients’ anxiety levels regarding third molar surgery. Furthermore, neither the duration since recovery from COVID-19 nor the highest body temperature recorded during infection was found to impact the dental anxiety toward the third molar surgery. It is encouraging to observe that, despite enduring the strict lockdowns measures and the rapid spread of the virus, Chinese people who were infected with COVID appears to have maintained their mental and physical well-being.

As reported by the COVID-19 Mental Disorders Collaborators, the psychological impact of the COVID-19 pandemic led to the elevation in the prevalence of multiple psychiatric symptoms, including major depression, anxiety, as well as other disorders (Daly and Robinson, 2022). However, the evidence-based study pointed out that the increase in mental health symptoms was temporary at the onset of the pandemic. The prevalence of symptoms could be decreased significantly over time and become indistinguishable from prepandemic symptom profiles within a few months (Robinson et al., 2022). As reported by Nicotra et al. (2023), the existence of a label for post-COVID-19 cognitive syndrome or psychiatric disorders was still discussed, and the objective evaluation of the symptoms is not standardized currently. In the present study, we aim to evaluate the variation of patients’ anxiety levels toward third molar extraction following the recovery from COVID-19. Given the profound impact of the ongoing public health crisis, we hypothesize that Chinese individuals who have endured COVID-19 infection may have undergone a range of psychological changes. These changes could potentially influence the level of anxiety experienced by individuals regarding dental treatments. Our hypothesis suggests that the MDAS scores will be elevated just after the SARS-CoV-2 infection, and with the passage of time, it might decline. However, the results of the present study did not find any significant change in patients’ MDAS scores before and 1 to 6 months after the infection and recovery of COVID-19, represented that the anxiety of patients toward third molar surgery might not be influenced by the Covid-19 infection. Additionally, the MDAS scores of patients who recovered 1–2 months after the infection were the lowest among the groups. These results could be explained by that people who had the courage to go to the hospital to receive a third molar surgery only 1–2 months post-COVID-19 might be less anxious or less frightened of the surgery at the beginning.

Fever is one of the typical and common symptoms of COVID-19. It was reported by Guan et al. that fever was found in 42.8% of the patients at the time of admission, and 88.7% at the time of hospitalization (Guan et al., 2020). Sung et al. (2023) found that patients with abnormal body temperature during COVID-19 quarantine were more likely to experience anxiety. Additionally, as the study conducted by Sykes et al. (2021) described, in the post-COVID stage, the presence of residual symptoms including anxiety, fatigue, and myalgia was not associated with the severity of the COVID-19 illness. In the present study, we investigated the association between the highest body temperature during COVID-19 and the MDAS toward third molar surgery. As a result, we failed to find a significant difference between the groups. However, we could observe from the outcomes that patients with a temperature < 37°C got the lowest MDAS score and patients with a temperature > 40. The significant results might be shown after the sample size accumulated, therefore further large sample-sized study is still needed.

Notably, the study revealed that female patients exhibited significantly higher MDAS scores compared to male patients (12.29 ± 4.53 vs. 9.91 ± 3.80, *p* < 0.001). This gender disparity in anxiety levels is consistent with previous research indicating that women generally report higher levels of anxiety compared to men (Dereci et al., 2021). Various factors, including hormonal differences, social factors, and psychological responses to pain and medical procedures, could contribute to this observed gender difference in dental anxiety (Malvania and Ajithkrishnan, 2011).

Moreover, patients who underwent extraction of double teeth reported significantly higher MDAS scores than those who had a single tooth extracted (12.03 ± 4.74 vs. 11.24 ± 4.18, *p* = 0.037). To the extent of our knowledge, this is the first time the relationship between anxiety and the number of the third molars to be extracted. The increased anxiety in patients facing double tooth extractions can be attributed to the anticipation of more complex surgical procedures, increased pain, and longer recovery times. The fear of more extensive dental work can exacerbate preoperative anxiety, reflecting the relationship between the perceived severity of the procedure and anxiety levels.

The study also identified a negative correlation between MDAS scores and age (*p* = 0.025), suggesting that younger patients are more likely to experience higher levels of dental anxiety. This trend echoes findings from Cianetti et al., who reported a decline in the prevalence of dental fear/anxiety among children and adolescents as age increased (Cianetti et al., 2017). These findings noticed us that older individuals may have greater life experience and coping mechanisms to manage stress and anxiety. Furthermore, older patients might have had more dental experiences, which could reduce fear through familiarization with dental procedures.

The presented study however has limitations. The sample size was limited and was not equal for each group. The information provided by the patients was all self-reported, especially the results of the COVID-19 test and the highest body temperature, which might lead to reporting bias and recalling bias. Besides, the socioeconomic, psychosocial characteristics of the patients were not recorded, which will influence anxiety. Moreover, the statistical analysis was not adjusted for other risk factors that may influence questionnaire scores.Therefore, it must be made more cautiously for the interpretation of results. Further prospective studies with balanced group designs and more comprehensive information collecting were still needed.

## Conclusion

In conclusion, the present study did not establish a significant impact of SARS-CoV-2 infection on the anxiety levels associated with third molar surgery among Chinese patients. The potential long-term biopsychological effects of the virus warrant further investigation. Future research should focus on longitudinal studies to monitor the persistence and evolution of anxiety related to dental procedures over time in the context of SARS-CoV-2 (Gupta et al., 2022). Besides, understanding that certain demographic groups, such as younger patients and women, are predisposed to higher levels of anxiety can help dental professionals tailor their patient management strategies. Enhanced communication, psychological interventions, and personalized care can be implemented to alleviate anxiety, especially for patients identified as being at higher risk.

## Data availability statement

The original contributions presented in the study are included in the article/supplementary material, further inquiries can be directed to the corresponding authors.

## Author contributions

JZ: Methodology, Project administration, Software, Validation, Writing – original draft, Writing – review & editing. WL: Conceptualization, Data curation, Formal analysis, Writing – review & editing. FW: Writing – review & editing. DZ: Writing – review & editing. MW: Investigation, Writing – review & editing. HZ: Investigation, Writing – review & editing. YZ: Writing – review & editing.
